# Human Endogenous Retrovirus K106 (HERV-K106) Was Infectious after the Emergence of Anatomically Modern Humans

**DOI:** 10.1371/journal.pone.0020234

**Published:** 2011-05-25

**Authors:** Aashish R. Jha, Douglas F. Nixon, Michael G. Rosenberg, Jeffrey N. Martin, Steven G. Deeks, Richard R. Hudson, Keith E. Garrison, Satish K. Pillai

**Affiliations:** 1 Department of Human Genetics, University of Chicago, Chicago, Illinois, United States of America; 2 Division of Experimental Medicine, University of California San Francisco, San Francisco, California, United States of America; 3 Albert Einstein College of Medicine, Bronx, New York, United States of America; 4 HIV/AIDS Division, Department of Medicine, San Francisco General Hospital, University of California San Francisco, San Francisco, California, United States of America; 5 Department of Evolution and Ecology, The University of Chicago, Chicago, Illinois, United States of America; 6 Department of Biology, Saint Mary's College of California, Moraga, California, United States of America; 7 Division of Infectious Diseases, Department of Medicine, San Francisco Veterans Affairs Medical Center, University of California San Francisco, San Francisco, California, United States of America; Harvard University, United States of America

## Abstract

HERV-K113 and HERV-K115 have been considered to be among the youngest HERVs because they are the only known full-length proviruses that are insertionally polymorphic and maintain the open reading frames of their coding genes. However, recent data suggest that HERV-K113 is at least 800,000 years old, and HERV-K115 even older. A systematic study of HERV-K HML2 members to identify HERVs that may have infected the human genome in the more recent evolutionary past is lacking. Therefore, we sought to determine how recently HERVs were exogenous and infectious by examining sequence variation in the long terminal repeat (LTR) regions of all full-length HERV-K loci. We used the traditional method of inter-LTR comparison to analyze all full length HERV-Ks and determined that two insertions, HERV-K106 and HERV-K116 have no differences between their 5′ and 3′ LTR sequences, suggesting that these insertions were endogenized in the recent evolutionary past. Among these insertions with no sequence differences between their LTR regions, HERV-K106 had the most intact viral sequence structure. Coalescent analysis of HERV-K106 3′ LTR sequences representing 51 ethnically diverse individuals suggests that HERV-K106 integrated into the human germ line approximately 150,000 years ago, after the emergence of anatomically modern humans.

## Introduction

Endogenous retroviruses (ERVs) are the fossilized, germ line-integrated remnants of exogenous retroviruses and comprise approximately 8% of the human genome [1], [2]. Upon integration into germ cells, ERVs are transmitted in a Mendelian fashion, accumulating mutations over time that erode and ultimately eliminate their original viral sequence structure. Just as ERV genomes are subjected to modification within the host, host-ERV interactions may also induce changes in the host genome. For example, homologous recombination between distant HERV loci have likely induced chromosomal rearrangements in the human host, and coevolutionary conflicts with the exogenous precursors of HERVs may have resulted in sequence modifications in human antiretroviral genes such as APOBEC3H and TRIM5α [3], [4], [5]. Thus, identifying HERVs that integrated into the human genome in the recent evolutionary past may allow us to catalog proviruses that influenced the evolution of genes involved in antiviral responses. In addition, HERVs have been implicated in playing a role in a number of diseases [6], [7], [8], [9], [10], [11]. It has been suggested that cross-talk between HIV-1 and HERV transactivation pathways may compromise HIV-1 eradication strategies aimed at purging latently-infected cellular reservoirs [12]. Thus, detailed investigation of the genomic architecture and evolutionary history of the most recent HERV insertions may provide vital insights into viral inactivation and the present-day conflict between humans and infectious retroviruses such as HIV-1 and HTLV-1.

The most ancient HERVs are 60–70 million years old [1], [13]. However, some members of the HERV-K family integrated into the human genome after the hominid-chimpanzee split 6 million years ago (Mya) [14], [15], [16], [17], and these human-specific insertions are considered the youngest of all ERVs in the human genome. In this study, we analyzed all full-length HERV-K (HML-2) loci in the human genome to determine how recently in human evolutionary history the infectious progenitors of endogenous retroviruses were active.

## Results

The human genome consists of numerous HERV insertions that contain 5′ and 3′ LTRs and varying remnants of the viral coding genes in between. Many more insertions currently exist as solo-LTRs, which are the remnants of full-length insertions that have been truncated into a single LTR by virtue of inter-LTR recombination events. Inter-LTR comparison is the standard method used to estimate the age of full-length HERV insertions. At the time of insertion, the LTR sequences are presumed to be identical and the differences between the 5′ and 3′ LTRs of the same provirus are believed to arise due to substitutions accumulating in a clocklike manner post-insertion [18]. To elucidate the insertion timeline of the human-specific endogenous retroviruses into the human genome, we obtained the complete genome sequences of all the human-specific full-length HERV-K insertion previously reported [15], [16] using the UCSC genome browser (hg18).

**Figure 1:**
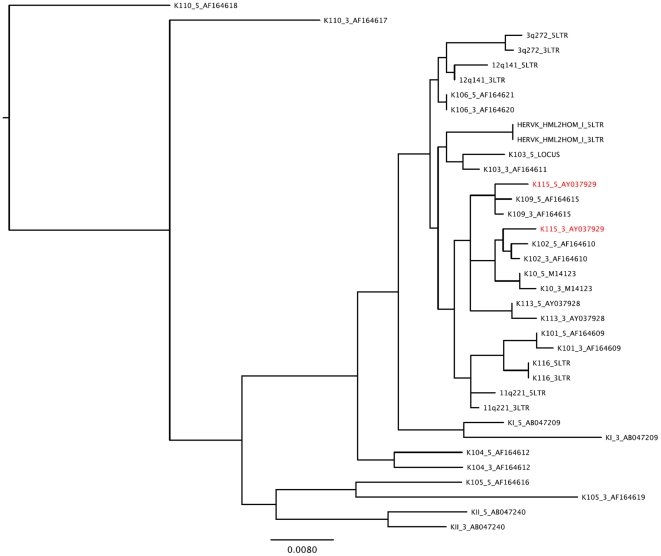
Phylogenetic tree of full-length HERV-K (HML-2) LTR sequences. The clustering of the HERV-K 5′ and 3′ LTR sequences of each insertion suggests that gene conversion is rare in the HERV-K family. Each HERV is indicated by its name (K106 for HERV-K106). HERV taxa that may have undergone gene conversion are indicated in red. HERV-K115 5′LTR clusters with HERV-K109 suggesting a gene conversion occurred between these two HERV-K members. HERV-K10, HERV-K HML2HOM, and HERV-K110 are sometimes referred to as HERV-K107, HERV-K108, and HERV-K18, respectively. HERV-K110 was used to root the phylogeny because it is present in both Humans and Gorillas [16].

It has previously been hypothesized that gene conversion may be rampant amongst HERV loci [19]. Pervasive gene conversion would prevent us from applying a molecular clock to these data, since observed sequence variation between LTR regions would be attributed to recombination rather than the stepwise accumulation of mutations predicted by the neutral theory of evolution [20]. We reconstructed a maximum likelihood phylogeny of all HERV-K LTRs to determine the prevalence of gene conversion between insertions in the HERV-K family (Figure 1). The clustering of the 5′ and 3′ LTR sequences associated with each insertion locus in our phylogeny suggests that gene conversion is rare among HERV-Ks, with the single exception of HERV-K115. Evidence of a gene conversion event in HERV-K115 has been reported previously [17], [19].

To estimate the insertion times of each human-specific endogenous retrovirus, we calculated the age of each human-specific full-length HERV-K insertion using the traditional inter-LTR comparison method [18]. Inter-LTR divergence measurements of the fourteen insertions that did not undergo gene conversion in the LTR region were converted to insertion age estimates by applying an established HERV-K LTR-specific divergence rate of 0.13% per million years (Myr) to these data [21] (Table 1). Based on this method, HERV-K1p31.1 (referred to as HERV-K116 from here onwards) and HERV-K106 have the highest probability of being the youngest full-length endogenous retroviruses in the human genome. Due to the perfect identity between their 5′ and 3′ LTR sequences, an inter-LTR comparison is only informative to calculate a maximum, upper-bound age estimate. A single mutation is predicted to arise within an LTR every 0.8 Myr based on the divergence rate of 0.13%/Myr. Having no sequence differences between their LTRs, inter-LTR analysis of HERV-K116 and HERV-K106 suggests that both insertions must be younger in age than the time required for one sequence polymorphism to arise between the two LTRs, or 0.8 Myr.

**Table 1:** Human specific complete HERV-K (HML-2) proviruses within the human genome.

S.No.	HERV	Accession no.	Location^1^	inter-LTR differences^2^	Average LTR size	Age^3^ (Myr)
1	K106	AF164620	3q13.2	0	960	<0.8*
2	K116^#^	N/A	1p31.1	0	968	<0.8*
3	K101	AF164609	22q11.2	2	968	1.6
4	K107	AF164613	5q33.3	2	968	1.6
5	K117^#^	N/A	3q27.2	3	968	2.4
6	K109	AF164615	6q14.1	3	960	2.4
7	K113	AY037928	19p13.11	3	968	2.4
8	K102	AF164610	1q21	4	968	3.2
9	K118^#^	N/A	11q22.1	4	968	3.2
10	K119^#^	N/A	12q14.1	5	974	3.9
11	K108	AF164614	7p22.1	6	968	4.8
12	K103	AF164611	10p12.1	7	968	5.6
13	K104	AF164612	5p14.3	12	964	12.0
14	K115	AY037929	8p23.1	13	964	N/A

Table 1 Notes

1 The identity and location of human specific complete HERV-K (HML-2) proviruses within the human genome were obtained from previous reports [15], [16]. **^#^**HERV-K116, K117, K118 and K119 were previously referred to by their genomic locations.

2 Three HERV-K members have as many differences as the insertionally polymorphic HERV-K113 and four HERV-K members have fewer differences between their LTR than HERV-K113 of which, HERV-K106 and HERV-K116 have identical LTR, however, the latter has a 2846 bp deletion in the *pol* region.

3 All age estimates are based on inter-LTR comparisons and are in million years (Myr). *Age estimates for HERV-K106 and HERV-K116 are based on 1 SNP between their LTRs. The age of K115 is listed as ‘N/A’ because it cannot be determined by inter-LTR comparison method. The age of K115 was previously estimated to be at least 1.1 Myr using coalescent approach [25].

**Figure 2:**
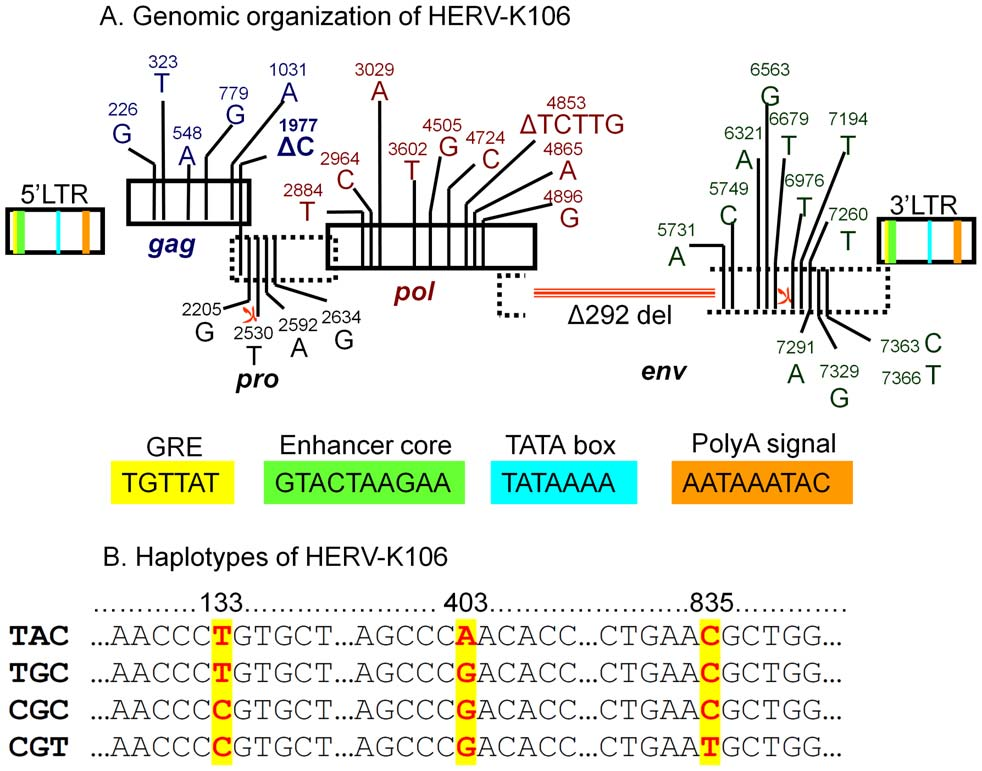
Genome organization and haplotypes of HERV-K106. Figure 2A: Genomic characterization of HERV-K106 demonstrating the two LTRs, gag, pol, and env genes. The HERV-K106 genome was annotated with the aid of the HERV-K consensus sequence (HERV-KCON) [23], two HERV-K HML2 members that have somewhat functional *gag* (HERV-K101 and HERV-K109) [43], and insertionally polymorphic HERV-K113 that has intact ORF [17]. Known functional elements within the HERV-K106 LTR are shown in colored boxes. All SNP positions in the coding genes are counted from the beginning of *gag* ORF. Five nonsynonymous SNPs shown in *gag* region are HERV-K106 specific. These SNPs do not include I516M mutation which singlehandedly eliminates functionality of HERV-K113 *gag* [43] warranting the future investigation of functionality of K106 gag. Although K106 lacks this vital SNP it harbors one base deletion at position 1977 which causes a frameshift near the end of the *gag* gene. Whether this frameshift causes gag to be dysfunctional is unknown. The HERV-K106 polymerase gene (*pol*) that includes reverse transcriptase is distinct from the other HERV-K family members used in our sequence comparison. In addition to the SNP shown, it harbors a 5 bp deletion from 4849–4853 bp that results in a frameshift mutation. The large 292 base deletion beginning at 5392 bp of pol extending into the env gene is the signature of type-I HERV-K [16]. In addition to the 292 bp deletion, HERV-K106 *env* gene has a premature stop codon. (<$>\raster(70%)="rg1"<$>) indicate stop codons in HERV-K106 genome. Figure 2B: SNPs and surrounding bases in 3′LTR of HERV-K106 demonstrating four haplotypes. Each haplotype is listed on the left, each SNP is represented in red and the position containing the SNP is highlighted in yellow.

We used Multalin [22] to align HERV-K113, K115, K106, and K116 with experimentally reconstituted infectious HERVs KCON [23] and Phoenix [24] that are based on artificial consensus sequences of full-length human-specific HERV-K (HML-2) insertions. We observed that while all four HERV insertions (K113, K115, K106, and K116) exhibited similarities to the reconstituted viruses, all four contained mutations that were unique to each insertion (Figure S1). We observed that both HERV-K106 and HERV-K116 are members of the type I HERV-K family as evidenced by the presence of a 292 bp ‘deletion’ in *env* which is the signature of all type I HERV members. The presence of this 292 bp *env* deletion in multiple HERV-K type I members suggests that this deletion may have been present in the infectious ancestral precursors of these viruses and probably does not render a HERV insertion dysfunctional on its own. However, HERV-K116 also has a 2846 bp deletion in its *pol* sequence [16]. In contrast, HERV-K106 exhibits relatively intact retroviral genome architecture (Figure 2A). Thus, these data suggest that HERV-K106 is the youngest endogenous retrovirus that survives largely intact in the human genome.

While inter-LTR comparison is a useful tool to estimate the age of HERV insertions, it becomes less informative with fewer mutations between the LTR regions and provides only an upper bound age estimate in the absence of sequence differences between the LTR regions. We recently developed an alternative dating method to infer insertion age when the inter-LTR method is inapplicable (to estimate the insertion dates of solo-LTRs and HERV loci such as K115 that show evidence of gene conversion) [25]. HERV insertions with identical 5′ and 3′ LTR sequences represent another scenario in which our alternative method is useful. We applied this method to the HERV-K106 insertion to derive a more precise estimate of its age. Our approach involves the application of coalescent inference to inter-host sequence variation in one of the proviral LTR sequences to estimate HERV insertion age. We generated complete HERV-K106 3′LTR sequences from 51 individuals representing various ethnicities and three different geographical locations within the United States (Table 2). PCR amplification and sequencing of the HERV-K106 LTR revealed three single nucleotide polymorphic positions (SNPs): 133, 403, and 835 (numbered according to their position in the GenBank reference sequence AF164620) (Table 2). Four HERV-K106 haplotypes could be constructed based on the SNPs identified in the 3′LTR region (Figure 2B).

**Table 2:** Base frequencies and haplotypes of HERV-K106 3'LTR with haplotype frequencies in various ethnic groups within the United States.

				Haplotypes of HERV-K106 3'LTR^2^					Haplotypes of HERV-K106 3'LTR by ethnicity^3^					
Base frequencies in HERV-K106 3'LTR^1^				base positions			Total n	Total f	Af. Am		Eur. Am		Others	
positions	Bases	n	f	133	403	835			n	f	n	f	n	f
133	C	83	0.88	C	G	C	80	0.85	20	0.77	40	0.91	20	0.83
	T	11	0.13											
				T	G	C	7	0.07	2	0.08	2	0.05	3	0.13
403	G	90	0.96											
	A	4	0.04	T	A	C	4	0.04	2	0.08	2	0.05	0	0.00
														
835	C	91	0.97	C	G	T	3	0.03	2	0.08	0	0	1	0.04
	T	3	0.03											
	**Total**	**94**				**Total**	**94**		**26**		**44**		**24**	

Table 2 Notes

1 Alleles at all three SNP sites in HERV-K106 3′ LTR consisted of two alternate nucleotide bases with one being predominant than the other (133: C>T, 403: G>A, and 835: C>T). Using these SNP four haplotypes of HERV-K106 could be constructed.

2 The most common haplotype was C-G-C (f = 0.90) followed by T-A-C (f = 0.05), C-G-T (f = 0.02) and T-G-C (f = 0.02).

3 77% of African American and 91% European Americans in our study had CGC haplotype of HERV-K106 LTR. CGC was also the most prevalent in a heterogeneous sample labeled as ‘others’ that consisted of a few (n<5 from each group) individuals of Hispanic, Asian, and East Indian origins. Haplotypes TGC and TAC were present in 8% and 5% of African Americans and European Americans respectively whereas haplotype CGT was present in 5% of African Americans but was absent in European Americans. African Americans, although in lower numbers in our study, demonstrated greater haplotype diversity than European Americans which is consistent with the “out of Africa” hypothesis that HERV-K106 originated in Africa and migrated out of Africa with human migration.

The coalescent estimation of insertion age rests on the assumption that the insertion site is evolving neutrally. Therefore, we conducted tests on the HERV-K106 insertion site to determine whether the assumption of neutrality was maintained. We performed 10,000 coalescent simulations using MS software [26] and Schaffner's calibrated model of human genome evolution [27] to calculate the probability of observing exactly 3 SNPs in a neutrally evolving, 960 bp stretch of human DNA. We assumed no recombination, and an inferred human mutation rate of 9.0×10^−9^ subsitutions per site per generation. These simulations yielded a Gaussian mutational probability distribution with a mean of 8 SNPs, and revealed that the presence of only 3 SNPs in a 960 bp neutrally evolving region of the human genome deviates significantly from expectations based on Schaffner's model (p<0.05). These findings suggest one of two possibilities about HERV-K106. Either it is evolving under selection, or it is evolving neutrally but has not evolved in tandem with the human genome for a sufficient amount of time to conform to the predictions of Schaffner's human genome-based model. We explored the selection hypothesis by performing standard tests of neutrality on the K106 locus, including Tajima's D [28], and Fu and Li's D* and H [29] (performed using DNASP [30]). In all cases, we could not reject the null hypothesis that HERV-K106 is evolving neutrally (p>0.10). HERV-K106 itself may have been evolving neutrally but could have been driven to fixation due to hitchhiking effects if the region flanking the insertion had been under positive selection. We used the HGDP Selection Browser [31] to test whether the genomic region containing the HERV-K106 insertion site is under selection by calculating the iHS and XP-EHH statistics on genotypes in the Human Genome Diversity Panel. The iHS and XP-EHH statistics are haplotype homozygosity-based tests used to detect signatures of recent selection on variants that have not yet reached fixation [32] and can be applied to detect selective sweeps in alleles that have approached fixation in one population but are polymorphic in the overall human population [33]. Even though HERV-K106 is fixed in all humans, the genetic region flanking the K106 insertion may contain SNPs that could reveal if a selective sweep has occurred in this region. We found that HERV-K106 is incorporated into a genomic location with only a few genes nearby (Figure S2) and the region in chromosome 3 containing HERV-K106 exhibited no signatures of selection, as both iHS and XP-EHH did not yield extreme values (Figure S3). These data collectively support that K106 is evolving neutrally and has only shared its evolutionary history with the human genome for a relatively short period of time.

We constructed a maximum likelihood phylogeny of all 94 observed HERV-K106 3′ LTR haplotype sequences to estimate the age of the K106 insertion (Figure S4). According to coalescent theory, the genetic distance to the inferred most recent common ancestor (MRCA) should reflect the time that has elapsed since the establishment of the ancestral sequence, and in this particular case, the age of the proviral insertion itself. We used two previously reported evolutionary rates to translate genetic distance into coalescence time. The upper-bound for the coalescence-based age estimate was inferred using the HERV-K LTR specific mutation rate of 1.3×10^−9^ mutations/site/year [21], and the inferred mammalian genome mutation rate of 2.2×10^−9^ mutations/site/year [34] was used to calculate a lower-bound estimate. Based on the two divergence rates, we estimate that HERV-K106 was integrated into the human genome between 91,000 and 154,000 years ago, after the emergence of anatomically modern humans [35], [36].

## Discussion

Our study suggests that HERV-K106 is a recent acquisition in the human genome and is possibly specific to *Homo sapiens sapiens*. HERV-K106 is fixed in humans, but HERV-K113 and HERV-K115 remain insertionally polymorphic [17], despite being inserted into the human genome several hundreds of thousands of years before K106 [25]. Features associated with the three regions of the human genome harboring these HERV insertions may help to explain the apparent discordance between insertion age and fixation. The fixation probability of an ERV may be inversely correlated with local chromosomal recombination rate and local gene density [37], [38]. We employed the high resolution recombination map of Kong et al. [39] to examine local recombination rates in the genomic neighborhoods of HERV-K106, K113 and K115 insertions. Analysis of the map revealed that HERV-K106 lies in one of only nineteen recognized “recombinaton deserts” in the human genome (crossover rates less than 0.3 c/Mb), which may have accelerated its fixation. Conversely, K113 and K115 reside in regions that are considerably more recombinogenic (1.18 cM/Mb and 2.14 cM/Mb, respectively) [39]. The low rate of recombination in the region surrounding K106 may have accelerated its fixation, or the high rates of recombination in the regions surrounding K113 and K115 may have decelerated the fixation of these older insertions. We next employed the NCBI Human Genome Map Viewer [40] to investigate the potential role of gene density in the neighborhood of these three HERV insertions, since a highly significant inverse correlation between local gene density and HERV fixation rate has been demonstrated [37]. The insertionally polymorphic HERV-K113 resides in chromosome 19, which has the highest gene density of all human chromosomes. We surveyed regions within a window of 1 Mb upstream to 1 Mb downstream of each HERV insertion for the presence of characterized genes to obtain a more localized estimate of gene density. HERV-K106, K113 and K115 are flanked by 32, 64, and 115 known genes, respectively. Again, the low gene density in the region surrounding K106 may have accelerated its fixation, or the higher gene densities in the regions surrounding K113 and K115 may have hindered the fixation of these older insertions. Taken together, the patterns of local recombination rate and gene density support genomic context as a primary determinant of fixation that explains the discordance between insertion age and insertional polymorphism in the human population observed within the HERV-K family.

**Figure 3:**
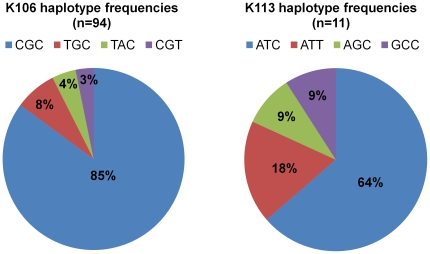
Comparison of haplotype frequencies between HERV K106 and HERV-K113. Haplotypes and haplotype frequencies of HERV K113 and K106 from individuals in the same sample set are shown. Haplotypes and haplotype frequencies of K113 were obtained from our previous study [25]. Higher minor haplotype frequencies (MHFs) were observed for K113 (18%, 9%, 9%) compared to K106 (8%, 4%, 3%) even though the numbers of K113 LTRs sequenced was much smaller than that compared to K106. Higher K113 MHFs in a subset of our samples compared to that of K106 suggests that K106 integrated into the human genome much later than K106 such that MHFs of K106 have not had enough time to reach higher frequencies in global populations. This serves as an additional evidence for recent insertion integration time of HERV-K106.

There are many fixed and insertionally polymorphic HERV-K(HML-2) solo-LTRs in the human genome [16], [41], [42] and it is possible that some of these unfixed solo-LTRs may have been derived from proviral insertions that are younger than HERV-K106. However, the absence of a second LTR from these insertions prevents us from including them in our current investigation because we relied on the inter-LTR comparisons to identify HERVs that recently integrated in the human genome. A second caveat stems from the reliance of our age estimates on previously established mutation rates; changes in local mutation or recombination rate may influence the apparent age of this insertion. However, our analyses of the local recombination rates surrounding the K106 and K113 insertions suggest that this is not likely to be a confounding factor. Furthermore, we previously hervotyped a small subset of individuals in our samples (n = 16) to study haplotype diversity in HERV-K113 [25]. Haplotype diversity for K113 in this smaller subset of samples was higher than the haplotype diversity that we observed for HERV-K106 in the same subset, as would be expected when comparing an older to a younger insertion. The frequencies of the K113 minor haplotypes in individuals of both African and European descent were much higher than the frequencies of minor haplotypes that we observed for HERV-K106 (Figure 3).

To the best of our knowledge, this is the first rigorous evidence that the infectious progenitors of human endogenous retroviruses were active after the origin of anatomically modern man, *Homo sapiens,* in Africa [35], [36].

## Materials and Methods

### Samples and DNA extraction

Peripheral blood samples were obtained with written consent from a total of 51 individuals drawn from the pediatric HIV cohort at Jacobi Medical Center (Bronx, NY), the SCOPE cohort of chronic HIV-1 infection (San Francisco, CA), and from blood donors to the Stanford Blood Bank (Palo Alto, CA). Twelve participants were perinatally HIV-1 infected children followed at Jacobi Medical Center, Bronx, NY. Twenty-five samples of HIV-1 infected adults were obtained from the SCOPE cohort in San Francisco, California and fourteen individuals from Stanford Blood Bank were anonymous, healthy HIV-1 negative individuals. The study was approved by the Institutional Review Boards at Jacobi Medical Center and the University of California at San Francisco (UCSF). All participants self-identified their ethnicities. HERV-K106 hervotyping [25] and haplotype construction for this study was carried out without prior knowledge of the ethnicity of the participants. Genomic DNA was isolated according to the manufacturer's protocol using QIAmp DNA Blood Mini Kit (Qiagen, Valencia, California, USA).

### PCR and gel electrophoresis

Insertion sites, solo-LTR, and full length HERV-K106 were detected using the HERV-K106 specific primers 5′-GGTGTTGCTGTGGAAGGTATTC-3′ and 5′-TCCATGGCTATCCACGAGA-3′ and the two step PCR protocol previously described [15], [16]. Neither insertional polymorphism nor solo-LTR formation was detected in our sample. We designed two primers HERV-K1063LTR1 5′-ATTTGGTGCCAGGAACTGAG-3′ and HERV-K1063LTR2 5′-AAGAAAAGGGGGAAATGTGG-3′ that were specific to the HERV-K106 3′LTR for sequencing purposes. HERV-K1063LTR1 annealed to the host flanking DNA upstream of HERV-K106 insertion in chromosome 3 and HERV-K1063LTR2 bound downstream of the 3′LTR to the *env* gene of HERV-K106. PCR was performed in a volume of 50 uL with 25 ng of genomic DNA in 1.0 uM of each primer, 200 uM of each dNTP, 25 mM of MgCl_2_, and 0.5 units of *Taq* DNA polymerase obtained from Applied Biosystems, Foster City, California, USA. PCR was conducted with 10 minutes of initial denaturing at 95°C, 30 cycles of 10 seconds at 94°C, 30 seconds at annealing temperature (61.5°C), and 59 seconds of elongation at 72°C followed by a 10 min final extension at 72°C. Amplified PCR products were stored at 4°C. The amplified PCR products were detected as ∼1200 bp bands in 2% agarose gel. A low-mass DNA ladder (Invitrogen, Carlsbad, California, USA) was used to verify the product size (Figure S5).

### Sequencing and sequence analysis

Sequencing was performed at MCLAB, South San Francisco using an ABI 3730XL sequencer. To determine an individual's hervotype (HERV genotype) for HERV-K106, sequences were aligned and single nucleotide polymorphisms (SNPs) were identified using Sequencher version 4.9 [25]. To rule out errors introduced during the PCR or sequencing process, PCR was performed twice on each genomic DNA sample and SNPs were verified by bidirectional sequencing of the amplicons produced by the two independent PCR on the genomic DNA. There were ten individuals who were heterozygous at the SNP sites. Six were heterozygous at only one site and the haplotypes of these six individuals could be inferred. The remaining four individuals were heterozygous at more than one position and were excluded from the analysis since their haplotypes could not be reconstructed. After excluding the ambiguous heterozygotes, we analyzed the base frequencies at the three SNP sites of the HERV-K106 3′LTR in 102 HERV-K106 sequences. The base frequencies and SNP positions are summarized in table 2. We also compared HERV-K106 diversity between various ethnicities in the USA represented in our sample. While African Americans (n = 13) and European Americans (n = 22) were abundant in our sample, other ethnic groups such as Asians, Hispanics, East Indians, and Native Americans were represented in very low numbers (n<5) in our sample. Thus, we compared HERV-K106 haplotype frequencies in African Americans and European Americans and the rest of the ethnicities were grouped together as ‘others’.

### Coalescence and phylogenetic inference

Coalescence time was calculated as described previously [25]. The sequence of the most recent common ancestor (MRCA) of the HERV-K106 insertion was inferred using maximum likelihood inference and K110 (GenBank Accession number AF164617) as an outgroup taxon (Figure S4). Coalescence (insertion) times were approximated using the formula 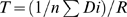 where T is the time (My) passed since the insertion event, *n* is the number of observed taxa, *D* is the divergence associated with a descendant taxon (cumulative branch lengths between observed haplotype and inferred MRCA), and *R* represents one of two previously reported divergence (evolutionary) rates (2.2×10^−9^ and 1.3×10^−9^ mutations/site/year) used to generate upper and lower bound estimates of insertion times [21], [34]. We used measurements of genetic divergence from inferred ancestral 5' LTR sequences (Figure S4) to estimate the HERV-K106 insertion time.

## Supporting Information

Figure S1 **Comparison of HERV-K106 genome to K113, K115, K116, KCON and Phoenix.** Multalin [22] was used to align K106 genome to that of two full length human specific HERV insertions (K113 and K115). We also included in the alignment two experimentally reconstituted HERVs KCON and Phoenix that are infectious and K116 that has a 2846 bp deletion in its pol gene. We observed that while all four HERV insertions (K113, K115, K106, and K116) exhibited similarities to the reconstituted viruses, all four contained mutations that were unique to each insertion. The identical regions in the genomes of all these HERV insertions are shown in blue. Genomic regions in red indicate regions that vary between at least one of these HERV insertions and ‘–’ indicates deleted regions.
(TIF)

Figure S2 **Genomic location of HERV-K106.** Top: High resolution genomic location of HERV-K106 showing insertion in a gene desert. No genes can be seen within ∼1 kb upstream or downstream of HERV-K106. Bottom: In a lower resolution genomic location map of the K106 insertion, the immune genes such as CD200R1 and CD200RL1 are visible far upstream of HERV-K106.
(TIF)

Figure S3 **Lack of selective sweep on or near HERV-K106 region.** The fixation of HERV-K106 in the human population could be due to drift, selection on HERV-K106, or a hitchhiking effect driven by other gene(s). If HERV-K106 reached fixation because it is under selection or due to a hitchhiking effect (i.e., if selection drove a nearby mutation to fixation and that mutation happened to be on a haplotype containing HERV-K106), then the XP-EHH statistic which measures complete selective sweeps should indicate selection acting on or nearby HERV-K106. We searched for signals of selection as measured by iHS and XP-EHH statistics in the samples from Human Genome Diversity Panels using tools on Prof. Johnathan Pritchard's webpage (http://hgdp.uchicago.edu/). We were unable to detect any signals of selection on or near HERV-K106. We were able to detect signals of selection in regions 500 kb farther away from HERV-K106 insertion region, indicating that selection can be detected in that region but HERV-K106 is not under positive selection.
(TIF)

Figure S4 **HERV-K106 3′LTR haplotypes.** ML tree of four haplotypesof HERV-K106 3′LTR. The most prevalent haplotypewas CGC (green) which is also the haplotypeof the HERV-K106 3′LTR in Genbank(AF164620). Minor haplotypes CGT (red), TGC (blue), and TAC (orange) are also shown. All haplotypescluster together. The HERV-K110 3′LTR was used as the outgroup.
(TIF)

Figure S5 **HERV-K106 3′LTR amplification.** The two primers specific to HERV-K106 3′LTR that we designed (HERV-K1063LTR1 5′-ATTTGGTGCCAGGAACTGAG-3′ and HERV-K1063LTR2 5′-AAGAAAAGGGGGAAATGTGG-3′) were used to amplify the complete HERV-K106 3′ LTR as detected by ∼1200 bp bands in 2% agarosegel. Low DNA Mass ladder (Invitrogen, Carlsbad, California, USA) was used to verify the product size.
(TIF)
