# Supplementary figures and images for: Human Endogenous Retrovirus K106 (HERV-K106) Was Infectious after the Emergence of Anatomically Modern Humans
Source: PLoS One. 2011 May 25;6(5):e20234. doi: 10.1371/journal.pone.0020234 (PMC3102101; doi:10.1371/journal.pone.0020234)

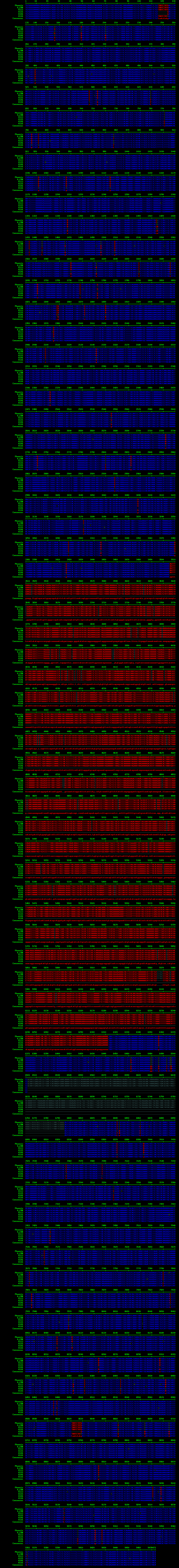

Supplement: Figure S1 — Comparison of HERV-K106 genome to K113, K115, K116, KCON and Phoenix. Multalin [22] was used to align K106 genome to that of two full length human specific HERV insertions (K113 and K115). We also included in the alignment two experimentally reconstituted HERVs KCON and Phoenix that are infectious and K116 that has a 2846 bp deletion in its pol gene. We observed that while all four HERV insertions (K113, K115, K106, and K116) exhibited similarities to the reconstituted viruses, all four contained mutations that were unique to each insertion. The identical regions in the genomes of all these HERV insertions are shown in blue. Genomic regions in red indicate regions that vary between at least one of these HERV insertions and ‘–’ indicates deleted regions. (TIF) [file pone.0020234.s001.tif]

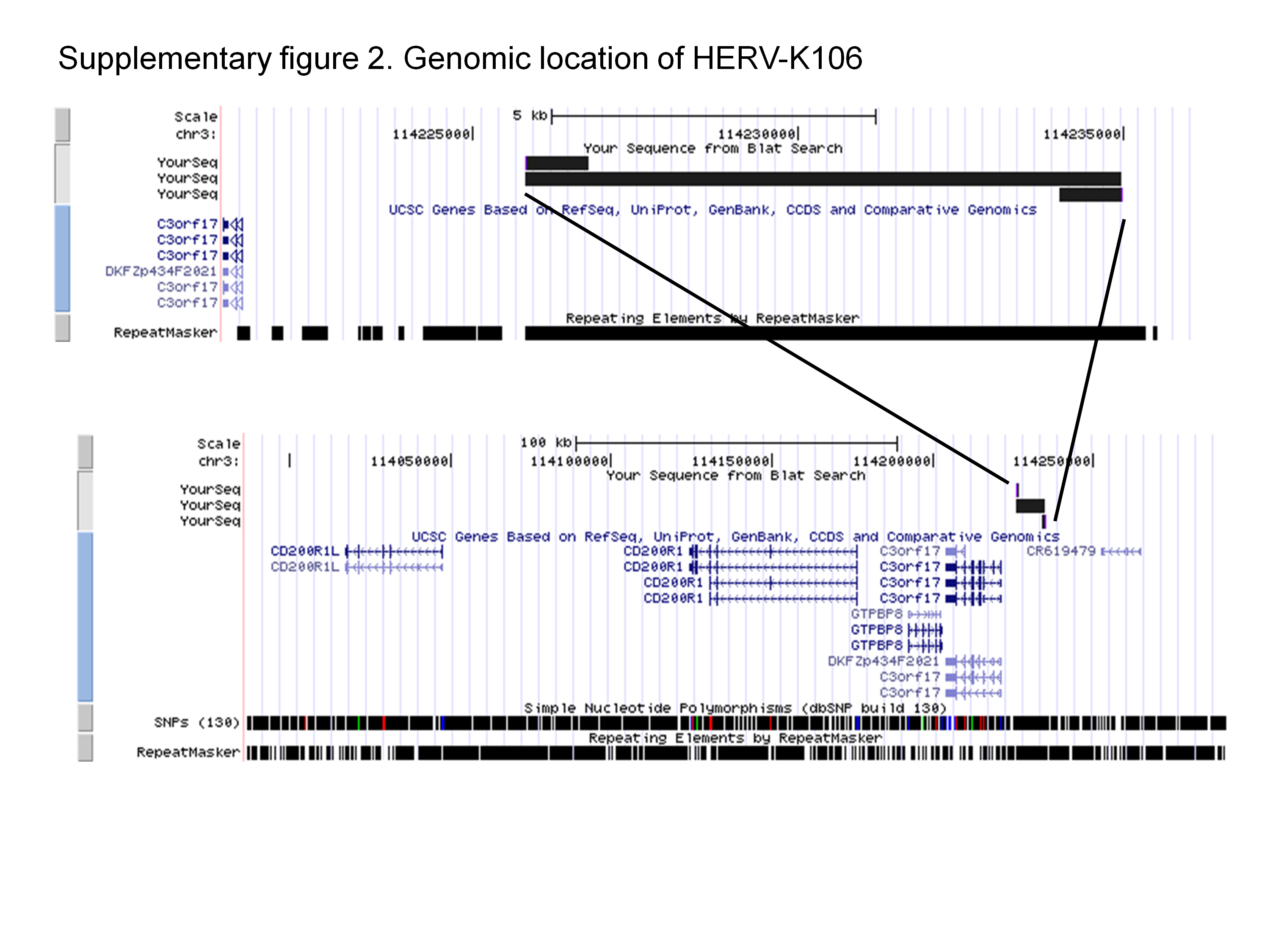

Supplement: Figure S2 — Genomic location of HERV-K106. Top: High resolution genomic location of HERV-K106 showing insertion in a gene desert. No genes can be seen within ∼1 kb upstream or downstream of HERV-K106. Bottom: In a lower resolution genomic location map of the K106 insertion, the immune genes such as CD200R1 and CD200RL1 are visible far upstream of HERV-K106. (TIF) [file pone.0020234.s002.tif]

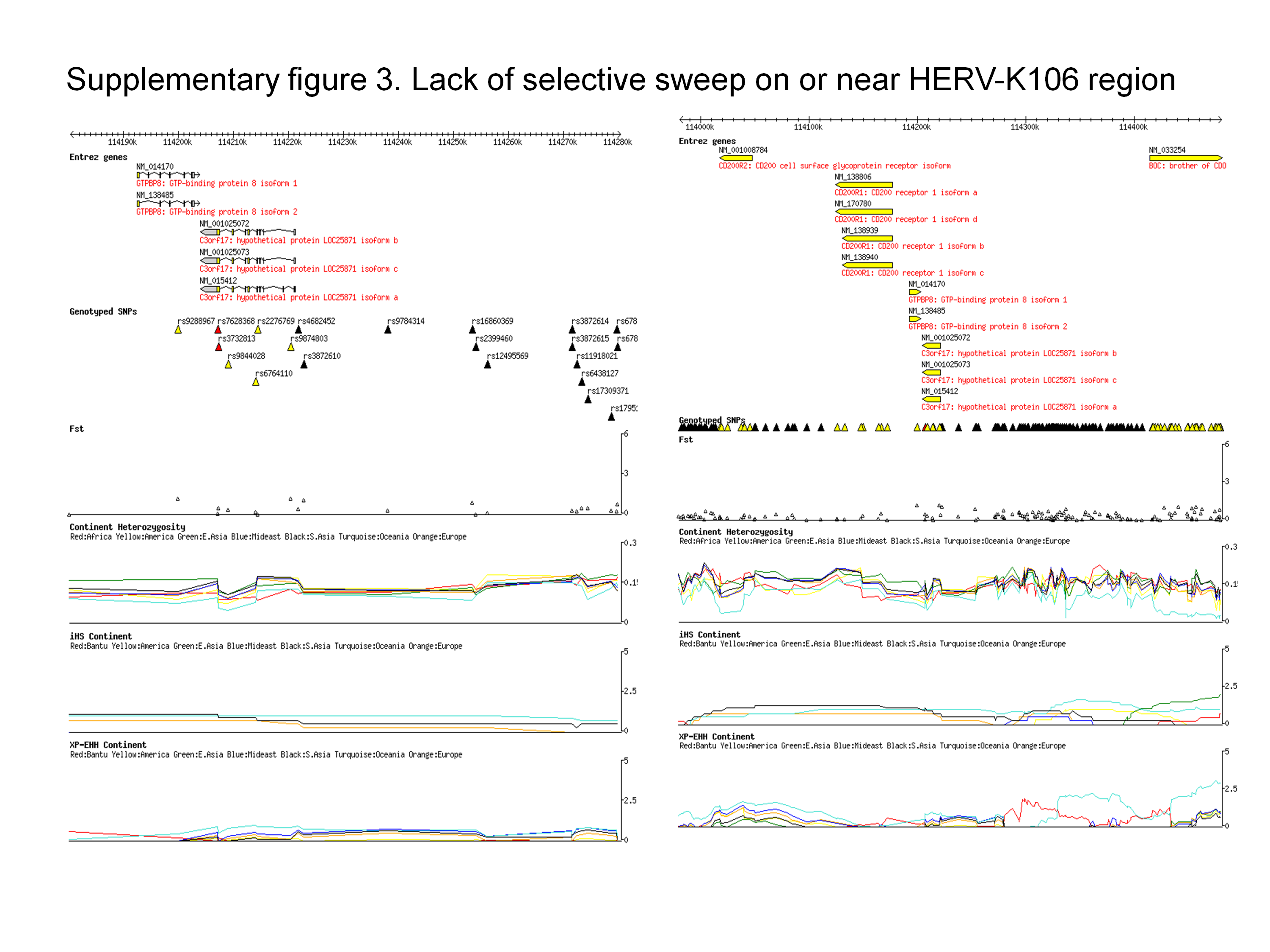

Supplement: Figure S3 — Lack of selective sweep on or near HERV-K106 region. The fixation of HERV-K106 in the human population could be due to drift, selection on HERV-K106, or a hitchhiking effect driven by other gene(s). If HERV-K106 reached fixation because it is under selection or due to a hitchhiking effect (i.e., if selection drove a nearby mutation to fixation and that mutation happened to be on a haplotype containing HERV-K106), then the XP-EHH statistic which measures complete selective sweeps should indicate selection acting on or nearby HERV-K106. We searched for signals of selection as measured by iHS and XP-EHH statistics in the samples from Human Genome Diversity Panels using tools on Prof. Johnathan Pritchard's webpage (http://hgdp.uchicago.edu/). We were unable to detect any signals of selection on or near HERV-K106. We were able to detect signals of selection in regions 500 kb farther away from HERV-K106 insertion region, indicating that selection can be detected in that region but HERV-K106 is not under positive selection. (TIF) [file pone.0020234.s003.tif]

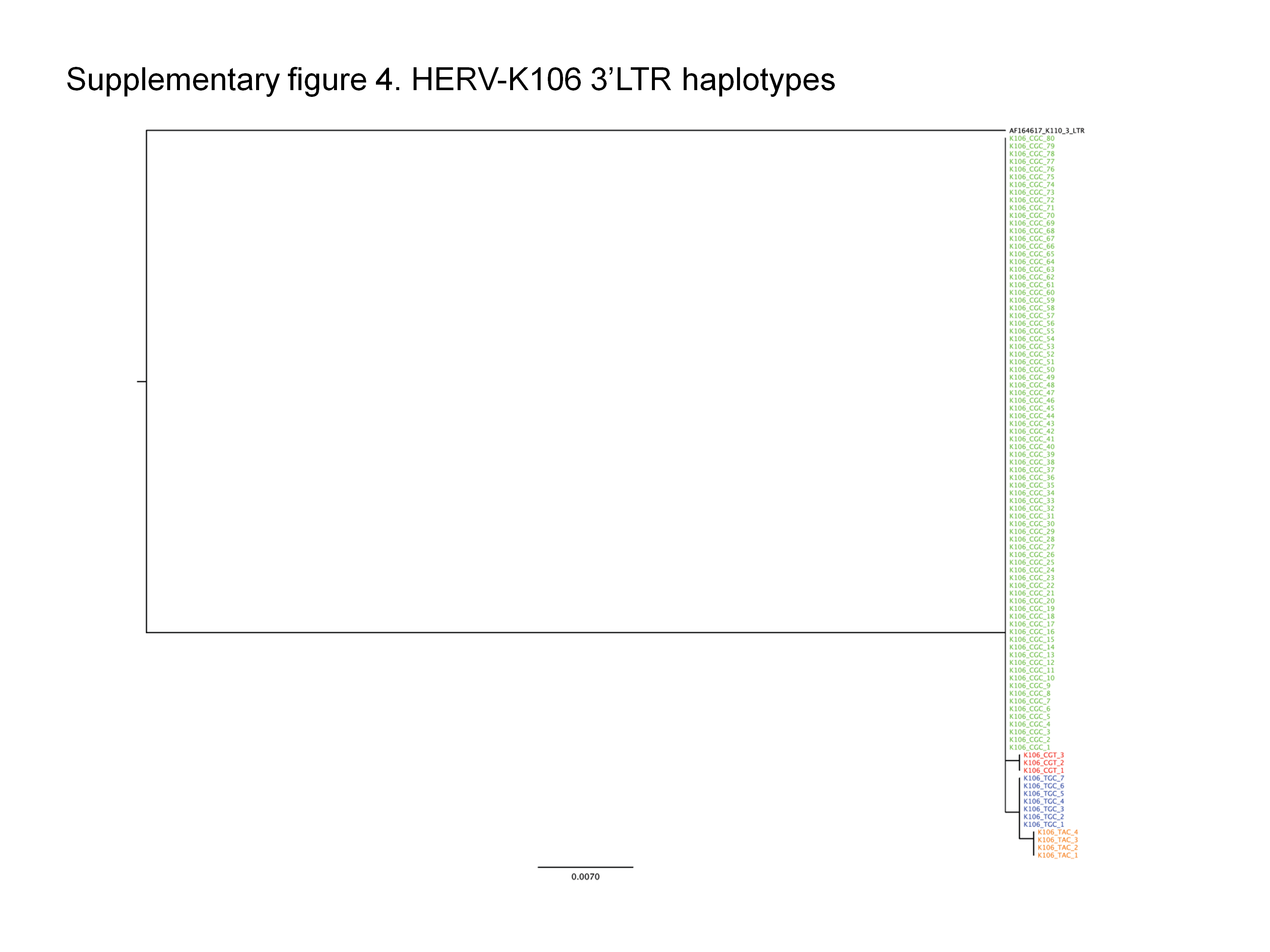

Supplement: Figure S4 — HERV-K106 3′LTR haplotypes. ML tree of four haplotypesof HERV-K106 3′LTR. The most prevalent haplotypewas CGC (green) which is also the haplotypeof the HERV-K106 3′LTR in Genbank(AF164620). Minor haplotypes CGT (red), TGC (blue), and TAC (orange) are also shown. All haplotypescluster together. The HERV-K110 3′LTR was used as the outgroup. (TIF) [file pone.0020234.s004.tif]

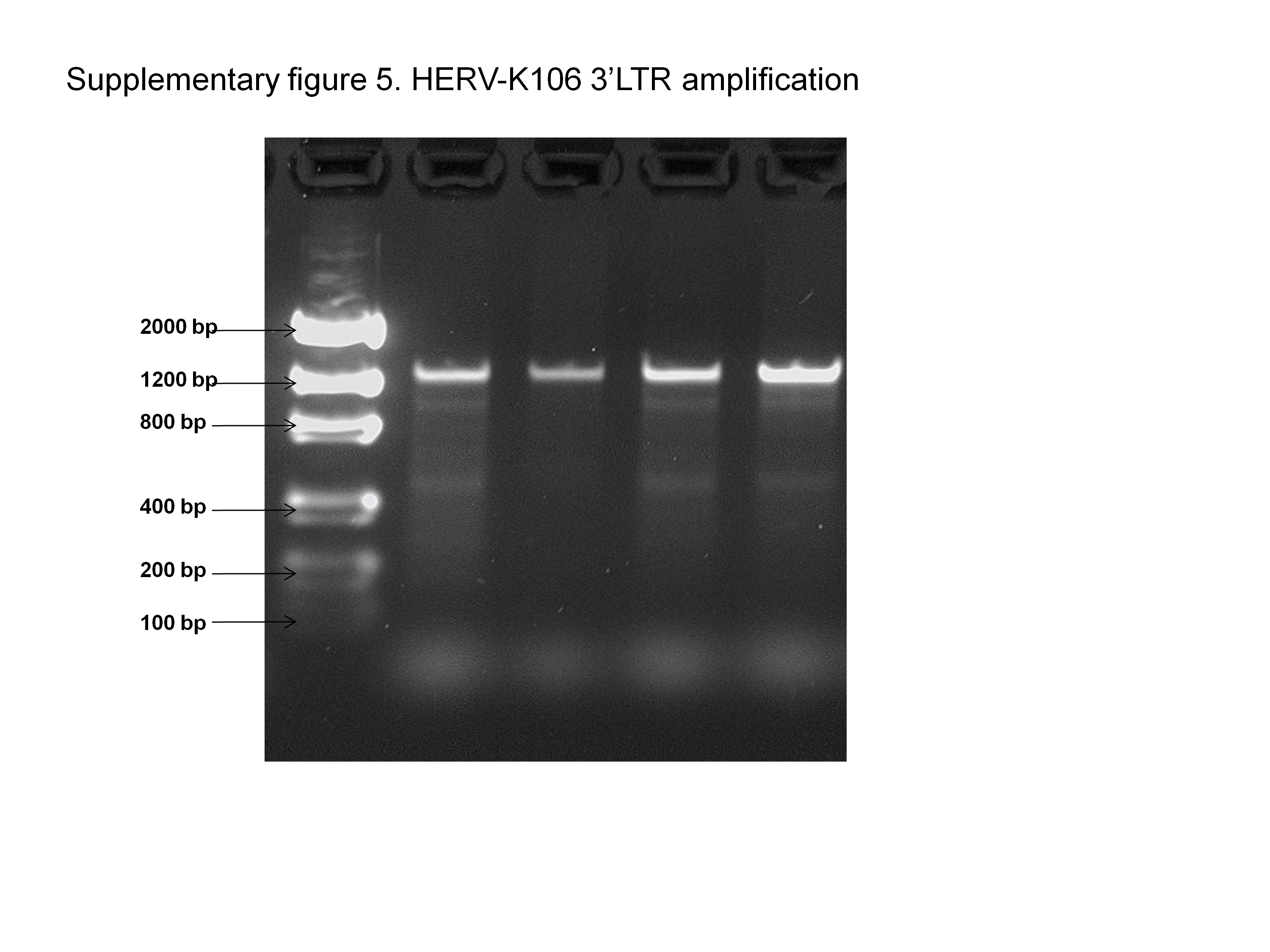

Supplement: Figure S5 — HERV-K106 3′LTR amplification. The two primers specific to HERV-K106 3′LTR that we designed (HERV-K1063LTR1 5′-ATTTGGTGCCAGGAACTGAG-3′ and HERV-K1063LTR2 5′-AAGAAAAGGGGGAAATGTGG-3′) were used to amplify the complete HERV-K106 3′ LTR as detected by ∼1200 bp bands in 2% agarosegel. Low DNA Mass ladder (Invitrogen, Carlsbad, California, USA) was used to verify the product size. (TIF) [file pone.0020234.s005.tif]
